# A High-Cholesterol Diet Increases Toll-like Receptors and Other Harmful Factors in the Rabbit Myocardium: The Beneficial Effect of Statins

**DOI:** 10.3390/cimb43020059

**Published:** 2021-07-26

**Authors:** Alkistis Kapelouzou, Michalis Katsimpoulas, Christos Kontogiannis, Irene Lidoriki, Georgios Georgiopoulos, Christos Kourek, Christos Papageorgiou, Konstantinos S. Mylonas, Spyridon Dritsas, Alexandros Charalabopoulos, Dennis V. Cokkinos

**Affiliations:** 1 Clinical, Experimental Surgery & Translational Research, Biomedical Research Foundation Academy of Athens, 11527 Athens, Greece; akapel@bioacademy.gr (A.K.); mkatsiboulas@bioacademy.gr (M.K.); 2 Attiko Hospital Animal, 19002 Athens, Greece; 3 School of Medicine, National and Kapodistrian University of Athens, 11527 Athens, Greece; kont_chr@hotmail.com (C.K.); georgiopoulosgeorgios@gmail.com (G.G.); chris.kourek.92@gmail.com (C.K.); chrispapageorgio@gmail.com (C.P.); 4 Vascular Unit, First Department of Surgery, Laiko General Hospital, National & Kapodistrian University of Athens, 11527 Athens, Greece; irenelido@gmail.com (I.L.); ksmylonas@gmail.com (K.S.M.); acharalabopoulos@yahoo.com (A.C.); 5 Second Department of Surgery, Aretaieio Hospital, National and Kapodistrian University of Athens, 11527 Athens, Greece; spdritsas@yahoo.com

**Keywords:** high-cholesterol diet, toll-like receptors, biomarkers, inflammation, statins, myocardium

## Abstract

Background: A high-cholesterol diet (HCD) induces vascular atherosclerosis through vascular inflammatory and immunological processes via TLRs. The aim of this study is to investigate the mRNA expression of TLRs and other noxious biomarkers expressing inflammation, fibrosis, apoptosis, and cardiac dysfunction in the rabbit myocardium during (a) high-cholesterol diet (HCD), (b) normal diet resumption and (c) fluvastatin or rosuvastatin treatment. Methods: Forty-eight male rabbits were randomly divided into eight groups (*n* = 6/group). In the first experiment, three groups were fed with HCD for 1, 2 and 3 months. In the second experiment, three groups were fed with HCD for 3 months, followed by normal chow for 1 month and administration of fluvastatin or rosuvastatin for 1 month. Control groups were fed with normal chow for 90 and 120 days. The whole myocardium was removed; total RNA was isolated from acquired samples, and polymerase chain reaction, reverse transcription PCR and quantitative real-time PCR were performed. Results: mRNA of TLRs 2, 3, 4 and 8; interleukin-6; TNF-a; metalloproteinase-2; tissue inhibitor of metalloproteinase-1; tumor protein 53; cysteinyl aspartate specific proteinase-3; and brain natriuretic peptide (BNP) increased in HCD. Statins but not resumption of a normal diet decreased levels of these biomarkers and increased levels of antifibrotic factors. Conclusions: HCD increases the levels of TLRs; inflammatory, fibrotic and apoptotic factors; and BNP in the rabbit myocardium. Atherogenic diets adversely affect the myocardium at a molecular level and are reversed by statins.

## 1. Introduction

The concept of atherogenesis has shifted from thrombotic to inflammatory and immunologic processes as possible pathogenetic factors [1]. Toll-like receptors (TLRs) are components of the innate immune system that respond to exogenous infectious ligands (pathogen-associated molecular patterns (PAMPs)) and endogenous molecules that are released during host tissue injury/death (damage-associated molecular patterns (DAMPs)) [2]. Interaction of TLRs with their ligands leads to activation of downstream signaling pathways which induce an immune response by producing inflammatory cytokines, type I interferons (IFNs) and other inflammatory mediators in the myocardium [3]. To date, 10 TLR members have been identified in humans and 12 TLR members have been identified in mice [4]. They play a determining role in the initiation and development of cardiovascular diseases.

We have previously shown that an atherogenic diet causes overexpression of TLRs 2, 3, 4 and 8 in rabbit aortas and that statins cause regression of this process [5]. In the current study, we assessed the effect of the same diet on myocardial TLR expression. We also examined the effect of this diet on inflammatory, pro- and antifibrotic and apoptotic factors and the brain natriuretic peptide (BNP) which is overexpressed in increased endomyocardial tension. Additionally, we evaluated the action of two commonly used statins, fluvastatin (Flu) and the more potent rosuvastatin (Ros), on these factors.

## 2. Materials and Methods

### 2.1. Animal Experimental Protocol

The experimental protocol was approved by the Animal Care and Use Committee of the Athens Prefecture Veterinarian Service, Greece (K/3319/4-5-2009). All experiments took place in the animal facilities of the Center of Experimental Surgery, Biomedical Research Foundation Academy of Athens (BRFAA), according to the guidelines set by the National Research Council’s Guide for Care and Use of Laboratory Animals.

The protocol of the study has been previously published and the samples referenced in this study derive from the 42 male (C90, C120, G30, G60, G90, GF120 groups) White New Zealand rabbits used in the study by Kapelouzou et al. [5] and 6 male (GR120 group) White New Zealand rabbits used in the study by Tziakas et al. [6], at the Biomedical Research Foundation of the Academy of Athens animal laboratory.

Following an acclimatization period of 1 week, 48 male White New Zealand rabbits (Trompetas Breeding Laboratories; Attiki, Greece) with an average body weight of 2.8 ± 0.2 kg were randomly divided into 8 groups of 6 animals each. High-cholesterol diet contained 1% cholesterol (2RB19; Mucedola, Milano, Italy). The protocol timeline of the study is described in Supplementary Figure S1. In the first experiment, 3 groups were fed with HCD for 1 (G30), 2 (G60) and 3 (G90) months. In the second experiment, 3 groups were fed with HCD for 3 months, followed by (a) normal chow for 1 month (G120) and (b) administration of fluvastatin (GF120) or rosuvastatin (GR120) for 1 month. Control groups were fed with normal chow for 90 (C90) and 120 (C120) days. Rosuvastatin (0.7 mg/kg BW) and fluvastatin (2 mg/kg BW) were administered daily by oral gavage. Control groups C90 and C120 were fed with normal chow for 90 and 120 days, respectively.

### 2.2. Tissue Preparation

At the end of the experimental period for each group, animals were euthanized with an intravenous overdose of sodium pentobarbital (100–120 mg/kg). The whole heart was removed and rinsed with distilled (DEPC-treated) water and shock-frozen to −140 °C for mRNA analysis.

### 2.3. mRNA Analysis

Total RNA was isolated from whole myocardium samples using the Tri Reagent (Sigma, Saint Louis, MO, USA), according to the manufacturer’s protocol [7]. All primers were synthesized by TIB Molbiol (Syntheselabor GmbH, Berlin, Germany). The sequences of primer pairs (Table 1) were designed with the Beacon Designer V7.0 software (Premier Biosoft International, Palo Alto, CA, USA).

Total RNA was extracted from rabbit myocardium of all groups using the Trizol kit (Invitrogen, Life Technologies, New York, NY, USA). The concentration of RNA was measured by an ultraviolet spectrophotometer (Biomate 3, Thermo Fisher Scientific, Waltham, MA, USA). RNA/hexamer mix was performed in a total volume of 13 μL. The total RNA (1~10 μL) was mixed with DEPC water (3 μL) and denatured at 70 °C for 5 min followed by incubation on ice for 5 min. Reverse transcription was performed in a total volume of 25 μL. The RNase inhibitor (50 U/L) (1 μL), reverse transcriptase (M-MLV 200U) (1 μL), dNTP (10 mmol/L, 5 μL) and RT buffer 5× (5 μL) were added to the denatured RNA samples (13 μL) and incubated at 37 °C for 60 min. Polymerase chain reaction (PCR) was performed in a total volume of 25 μL containing 2 μL of reverse transcription products (cDNA), 0.5 μL of Taq polymerase (5 U/L), 2.5 μL of Thermo 10× buffer, 1 μL of dNTP (2.5 mmol/L), 17 μL DEPC water and 1 μL of each forward and reverse primers. The parameters for the PCR reaction of β-actin were 95 °C for 10 min, 95 °C for 1 min, 57 °C for 1 min and 72 °C for 1 min, followed by 35 cycles of 57 °C, 72 °C for 5 min and final extension 10 °C. For all biomarkers, the PCR reaction parameters were 95 °C for 10 min, 95 °C for 1 min, 57 °C for 1 min and 72 °C for 1 min, followed by 45 cycles of 57 °C, 72 °C for 5 min and final extension 10 °C. The PCR products were electrophoresed on a 1.5% agarose gel. The qRT-PCR was performed in a total volume of 20 μL containing 2 μL cDNA, 7 μL DEPC water, 1 μL of each forward and reverse primers and 10 μL of SYBR-Green. The parameters of qRT-PCR were for all genes 52 °C for 5 min, 95 °C for 2 min, 95 °C for 15 s and 59 °C for 40 s, followed by 50 cycles of 59 °C. The qRT-PCR products of the β-actin gene were used as an internal control, and the relative expression levels of all the genes were calculated according to the ΔΔCT method [7]. The 2^-^ΔΔCT method analysis of relative gene expression using qRT-PCR was used to calculate the relative changes in gene expression. All data were normalized by β-actin levels and expressed as percentages relative to controls, as previously described [8]. Polymerase chain reaction (PCR), reverse transcription polymerase chain reaction (RT-PCR) and quantitative real-time polymerase chain reaction (qRT-PCR) were performed with the automatic thermal cycler Multi Cycler PTC-200 (MJ Research Inc, South San Francisco, CA, USA). Data for quantitation of gene expression were collected using a Chromo4 RT-PCR detector and analyzed with the Opticon Monitor Continuous Fluorescence Detector 3 software (MJ Research Inc., South San Francisco, CA, USA).

### 2.4. Statistical Analysis

According to the power analysis for independent samples, the required sample size per group was 6 (power analysis = 0.95, alpha = 0.05, beta = 0.05). Data are presented as mean ± standard deviation (mean ± SD). One-way ANOVA was used for the statistical analysis between groups. ANOVA trend analysis was used for the comparison of the rise of all factors described. Subsequently, we implemented one-way analysis of variables with test for linear trend between mean and col. number. All statistical calculations were performed using GraphPad Prism version 4.03 (GraphPad Software, San Diego, CA, USA). Statistical significance was considered at *p* < 0.05.

## 3. Results

### 3.1. Toll-Like Receptors

**Figure 1:**
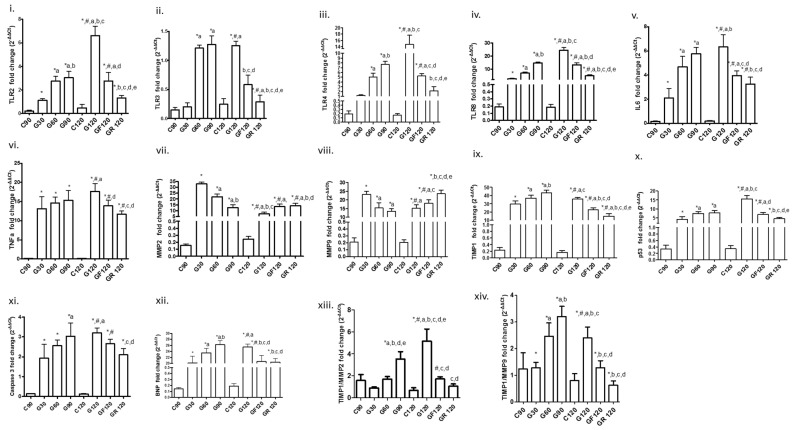
mRNA expression of biomarkers. TLRs 2 (**i**), 3(**ii**), 4 (**iii**) and 8 (**iv**); IL6 (**v**); TNFa (**vi**); MMPs 2 and 9 (**vii**,**viii**); TIMP1 (**ix**); p53 (**x**); CASP3 (**xi**); BNP (**xii**); ratio TIMP1/MMP2 (**xiii**); and ratio TIMP1/MMP9 (**xiv**) in myocardium. Significant difference (*p* < 0.05) versus C90 (*), C120 (#), G30 (a), G60 (b), G90 (c), G120 (d), GF120 (e).

A statistically significant increase was found in the mRNA expression as regards TLRs 2, 4 and 8 between C90, G30, G60 and G90; TLR3 also increased, but the increase started at a later time interval (G60) (Table 2 and Figure 1). A reduction in mRNA expression of TLRs 2, 3, 4 and 8 was found after Flu (GF120) and Ros (GR120) therapy compared to G120 during which TLRs continued to increase. Furthermore, we observed that TLR mRNA levels in GR120 decreased to a greater degree than in GF120.

### 3.2. Inflammatory Markers

Interleukin 6 (IL6) and tumor necrosis factor a (TNFa) increased in all atherogenic groups compared to control groups (Table 2 and Figure 1). Flu and Ros treatment decreased mRNA levels of IL6 and TNFa after the atherogenic diet compared to the G120 group (Table 2 and Figure 1), again with Ros exerting a stronger effect than Flu.

### 3.3. ΜΜP2, MMP9, Tissue Inhibitor of Metalloproteinase-1 (TIMP1) and Their Ratio

An elevation in both MMP2 and MMP9 mRNA expression was found in G30. Subsequently, a decrease was found in G60 and G90, although they both remained significantly elevated as compared to C90. They further increased at G120 and with both statins, Ros showing a higher increase in MMP9 at GR120 (Table 2 and Figure 1) than Flu (GF120). TIMP-1 increased progressively with HCD and decreased after resumption of a normal diet (G120); its expression further decreased with statins, to a greater extent with Ros. Besides these findings, we also observed that the TIMP1/MMP2 and TIMP1/MMP9 ratio increased significantly with HCD and decreased with statins (Table 2, Figure 1), again with Ros being more potent.

### 3.4. Apoptotic Factors

Apoptotic factors tumor protein 53 (p53) and CASP3 increased progressively in all HCD groups (Table 2 and Figure 1). With resumption of the normal diet (G120) a further increase in p53 and CASP3 mRNA expression was found (Table 2 and Figure 1). Both factors’ mRNA levels were lowered with statin treatment, to a greater extent in group GR120 than GF120 (Table 2 and Figure 1).

### 3.5. Biomarker of Myocardial Dysfunction

BNP mRNA expression consistently increased in HCD groups (Table 2 and Figure 1), and then it decreased in GF120 and GR120 compared to G120. No differences were found between the two statin treatment groups (Table 2 and Figure 1).

### 3.6. Trend of Biomarkers

**Figure 2:**
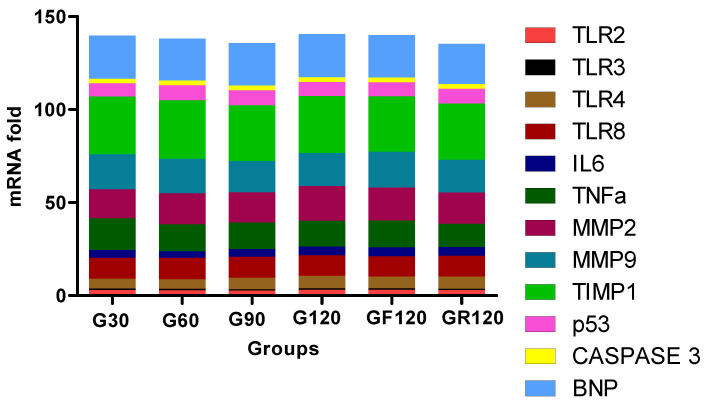
The trend of the biomarkers’ mRNA fold expression in HCD experimental groups.

Finally, we studied the mRNA expression trend of TLRs and other biomarkers. Figure 2 presents the mRNA fold expression of each biomarker in every group. We found that the TLRs have lower expression compared to the other inflammatory, apoptotic, MMP and dysfunction biomarkers.

## 4. Discussion

In this study, we diverged from the usual focusing on the atherosclerotic changes induced by an HCD in the arterial system, including the coronary arteries, and instead investigated its possible effects on the myocardium. In the great majority of these factors, a common pattern was seen: with continued HCD, TLRs and other noxious factors increased steadily, until the 90th day. Interestingly, this increase usually persisted throughout the 30 additional days after resumption of normal feeding (G120) but only started decreasing after statin treatment, with Ros, as a more potent statin, having a stronger effect than Flu.

### 4.1. TLR Overexpression

The two more abundant TLRs in the myocardium, TLR2 and TLR4, and the less prevalent TLR8 showed a similar course of increase with HCD, as already described. TLR3 showed a slightly different course, increasing later (at 60 days), possibly because it is situated in the endosomal compartment and not the membrane, thus being less promptly altered with HCD [4]. This was also shown by our group in the rabbit aorta [5]. TLR3 is an essential component of the innate stress response in virus-induced cardiac injury, with TLR3-/- mice showing less marked inflammatory changes [9]. It has an “unexpected” protective role in the arterial wall, potentially through a repair mechanism [10]. In the myocardium, Fattahi et al. [11] have found that it is involved in cardiac dysfunction developing during polymicrobial sepsis. Gao et al. [12] also found that it contributes to persistent autophagy and heart failure in mice after an experimental myocardial infarction, while it does not promote inflammation.

TLR8 is often lumped together with TLR7. In humans, Jurk et al. [13] postulate that it can activate gene transcription of NFkB. Salagianni et al. [14] have found that TLR7 has a protective antiatherosclerotic role by decreasing monocyte/macrophage proinflammatory activity. Triantafillou et al. [15] have shown that in the human myocardium, inflammatory responses are triggered by Coxsackie B viruses through TLR8 and to a lesser extent through TLR7. It recognizes viral or bacterial single-stranded RNA and activates innate immune systems and early inflammatory responses.

It has been well described that TLRs are involved in ischemia-induced inflammation [3,16]. It is unknown through which exact mechanisms high cholesterol levels can induce inflammation and TLR increase and activation of the cardiac immune response. However, TLRs are regulated by hypercholesteremia, hyperlipidemia and hyperglycemia [17]. Ruysschaert and Lanez [18] have shown that cholesterol and lipid microdomains influence TLR activity. Methe et al. [19] have found that statins decrease TLR4 expression and downstream signaling in human CD14+ monocytes.

Our finding that chronic HCD induces myocardial TLR overexpression has an important clinical corollary: since high-fat feeding also has been shown to induce severe coronary atherosclerosis, a clinical situation can emerge in which a myocardial infarct due to exacerbated coronary atherosclerosis can be precipitated in a high-TLR-expressing myocardium. This may have important unfavorable clinical effects: (a) TLRs exacerbate I/R injury [3,4,5,6,7,8,9,10,11,12,13,14,15,16]. Actually, TLR4 deficiency has been found to reduce myocardial infarction size [20]. The TLR4 specific antagonist eritoran reduced infarct size [21]. (b) TLRs contribute to postinfarct remodeling. Shishido et al. [22] found that TLR2-deficient mice developed less cardiac remodeling after myocardial infarction. Timmers et al. [23] found the same for TLR4-deficient mice. Frantz et al. [24] also showed that TLR4 is increased in the total failing myocardium.

As shown in a previous study from our group, statins can diminish TLR mRNA expression in the aorta [5]. Fluvastatin, simvastatin and atorvastatin have all shown anti-TLR4 activity [25]. We showed that Ros is more potent than Flu in this aspect. It also caused a greater reduction in plasma cholesterol [5]; thus, the question of whether cholesterol levels directly influence TLR expression remains. As regards the interaction of TLRs and apoptosis, Aliprantis et al. [26] have shown that bacterial lipoprotein (BLP) stimulates TLR2, which signals for apoptosis through MYD88 via Fas-associated death domain protein and caspase 8. They also found that BLP activates caspase 1 through TLR2. Ruckdeschel et al. [27] found that Yersinia infection can initiate apoptosis through TLR4 signaling.

### 4.2. Other Harmful Factors

The increase in IL6 in TNFa and HCD groups, indicating inflammation in the myocardium, is in accordance with the results of Ternacle et al. [28], which will be discussed later. Bartekova et al. [29] remark that circulating IL1b is elevated in dilated cardiomyopathy and associated with an adverse prognosis.

Interestingly, an opposite course with HCD was seen between the MMP2 and MMP9 collagen-degrading proteins and the profibrotic TIMP1, suggestive of increasing myocardial fibrosis. MMP2 and MMP9 increased very early (30 days) with HCD and declined thereafter, although remaining at much higher levels than control. However, they also started increasing again after statin treatment, with Ros showing a stronger effect than the generally weaker Flu. Fujimoto et al. [30] found increased vascular MMP deposition after a 4-month HCD following balloon de-endothelization of the abdominal aorta; this regressed after atherogenic diet withdrawal and Flu treatment. Galis et al. [31] found that MMP2 together with TIMP1 and TIMP2 were expressed by VSMCs in all layers of atherosclerotic arteries, while MMP1, MMP3 and MMP9 were localized to macrophages, VSMCs and endothelium in the fibrous cap and shoulder of the lesion. Ikeda et al. [32] also mention the increased expression of several MMPs, including MMP2 and MMP9, in the shoulder areas of plaques. In human atherosclerotic lesions, Orbe et al. [33] found an increase not only in MMPs but also in TIMP1 in calcification areas. Thus, the further increase in both MMPs with statin treatment may represent an adaptive phenomenon: MMPs may increase as an antagonistic response to TIMP1 increase. In fact, TIMP1 increased progressively in our HCD group, decreasing with resumption of a normal diet, but to a greater degree with statins, especially Ros. This factor is associated with the buildup of the fibrous plaque of coronary lesions with an increase in the TIMP1/MMPs ratio [34]. Actually, in our study, the TIMP1/MMP2 and TIMP1/MMP9 ratios decreased, implying an antifibrotic action with statin treatment. Both TIMP1 and MMPs are increased in the myocardium in dilated cardiomyopathy [35]; however, more information is needed. MMP2 structure and function are correlated with increased remodeling after an acute myocardial infarction [36]. MMP9 is also correlated with remodeling and mortality after an infarct [37]. MMP2 degrades troponin I in IRI ischemia–reperfusion injury [38], while in aortic stenosis, TIMP1 and TIMP2 are related to fibrosis [39]. TIMP1 also promotes myocardial fibrosis in pressure overload [40]. In 669 patients in the Framingham Heart Study, MMP9 was positively correlated to left ventricular mass and thickness and negatively to fractional shortening [41]. Moreover, serum MMP9 and TMP1 are also significant risk factors in population studies [42,43].

As regards apoptosis, the transcriptional factor p53 showed similar behavior to the already mentioned markers. It stimulates the proapoptotic protein bax [44]. Caspase 3, a main effector of apoptotic cell death, also showed a significant increase up to 120 days with HCD. Again resumption of a normal diet was not enough to cause a reversal of this trend, which was only seen with statin treatment, again with Ros being more potent than Flu. Dixon et al. [45] underline the role of caspase 1 in nonalcoholic steatohepatitis induced by a high-fat diet.

We report, for the first time, an increase in BNP in the myocardium with an HCD together with the other fibrotic, inflammatory and apoptotic factors. BNP is produced in increased quantities by the ventricular cardiomyocytes when endoventricular pressure is increased; it is also increased in ischemia, left ventricular hypertrophy and remodeling [46]. BNP is also produced by fibroblasts [47]. Interestingly, it also induces the expression of MMP2 and TIMP2, according to Tsuruda et al. [48]. It was interesting that all biomarkers showed a strong correlation in their increase. This is not surprising, since strong interactions exist among them in their rise and fall.

In accordance with our findings, Ternacle et al. [2] found that 4- and 20-week high-fat (13.5% fat) diets compromised myocardial function as expressed by radial strain rate, although the LVEF did not change, suggesting that novel, more advanced echocardiographic indices are needed to detect subtle myocardial function alterations. They also noticed an increase in myocardial fibrosis, inflammation, tissue oxidation and apoptosis. These authors ascribed these changes to obesity. Their animals (mice) increased in weight by around 40%, while ours only increased by 16%. With these numbers, we cannot ascribe our results to obesity, but rather to a direct action of the HCD.

Carbone et al. [49] fed mice for 4 weeks with a Western diet, i.e., a diet high in saturated fat and sugar. They found a decrease in LVEF and an impairment of diastolic function. Switching to standard diet, it was found that inhibition of the proinflammatory IL18 in mice fed a Western-type diet attenuated cardiac dysfunction despite a body weight gain of 38%. This finding also suggests that weight gain is not the determining factor in cardiac dysfunction. Resuming a normal diet for 4 weeks partially reversed this dysfunction. Carbone et al. [50], in another study, and Drosatos and Schulze [51] found that obesity-related cardiomyopathy and diabetic cardiomyopathy are caused by excess cardiac lipid accumulation. They describe that lipotoxicity can signal apoptotic pathways, and the treatment of neonatal rat ventricular myocytes with palmitic acid alters mitochondrial physiology, leading to apoptosis. It is interesting that both lipotoxicity and the more commonly studied myocardial glucotoxicity [52] do not only affect the myocardium. In fact, a lipotoxic model of pancreatic B cell failure has been produced, which involves histone modifications, linked to epigenetics [53]. Both hyperglycemic [54] and lipotoxic [55] cellular epigenetic memory can be established. The fact that normal diet resumption alone was not adequate for reversing molecular changes but statins were additionally needed may also suggest intensive treatment in hypercholesterolemic patients, especially if they are hypertensive and hyperglycemic, to avoid not only direct but also epigenetic changes. Thus, our study offers further evidence that an HCD not only affects the arterial system but also the myocardium. From the above, it can be seen that apart from our rabbit model, mice showed the same behavior [28,49].

Importantly, the role of statin administration in heart failure is intensively discussed. Lee et al. [56] have shown that pravastatin (a relatively weak statin) administration can cause regression of LV mass in hypercholesterolemic patients.

In a meta-analysis of 4500 patients from six studies, Deo et al. [57] showed that statins improve long-term survival in nonischemic cardiomyopathy. Gastelurrutia et al. [58], in a prospective study of 960 patients with both ischemic and nonischemic heart failure etiology, showed an improvement in mortality regardless of etiology. Two very experienced authors have raised the question of whether we need a large-scale outcome trial of statins in chronic heart failure [59]. Statins can induce important side effects, particularly in skeletal muscle, especially in aged rats [60]. However, the effects of statins on the cardiomyocytes have not been specifically reported.

### 4.3. Study Limitations

We believe that the main limitation of our study is that did not evaluate changes in cardiac function during HCD by echocardiographic or area–pressure measurements. Moreover, all our measurements were carried out in whole heart samples not differentiating between atria and ventricles. However, Yu et al. [61] and Nishimura et al. [62] also studied whole heart mRNA TLR expression. In human whole heart tissues, TLR2 and TLR4 are reasonably well expressed, but in a weaker concentration than in peripheral blood leukocytes [63]. However, we believe that our findings set the stage for further investigations on heart function in hypercholesterolemic patients and the effect of drastic lipid-lowering. The fact that TLRs can now be studied by ELISA in the serum [64] will make these studies easier and more applicable. Last, but not least, we did not perform histopathological analysis of the myocardium. However, Ternacle et al. [28] found myocardial inflammation, fibrosis, oxidation and apoptosis in their experiments in mice.

## 5. Conclusions

We showed that a high-cholesterol diet robustly increases the mRNA levels of TLRs, as well as inflammatory, fibrotic and apoptotic processes and BNP expression, in the whole rabbit heart, suggesting a direct effect of this diet on the myocardium. The changes in all these factors were not readily reversed by normal diet but were reversed with statin treatment, with Ros showing a more potent influence than Flu. A very strong correlation of these changes at all time intervals strengthens the significance of our findings.

Our findings strongly suggest that statin therapy may protect not only the arteries but also the myocardium, with one of the mechanisms involved being TLRs. This may be a timely finding in view of the newer trends towards achieving cholesterol levels as low as possible in patients with coronary artery and atherosclerotic vascular disease, since these efforts may also exert an additional favorable action on the myocardium.

## Figures and Tables

**Table 1:** Primers for real-time polymerase chain reaction (PCR) for each biomarker.

Gene	Forward	Reserve
TLR2	5′-CTCCTGCTGACGCTGCTC-3′	5′-TTCCTCGGCTTCCTCTTGG-3′
TLR3	5′-ATCTCCTCTCTTTGGGGACTGTTG-3′	5′-TGTTGGTGGGCAGGTCATCAGG-3′
TLR4	5′-CTCACATCCGAGTTGCCTTCCG-3′	5′-AAATGCTCCCTGGTACACCTGTTC-3′
TLR8	5′-ATCTTGTTCTTCTTCTCGTTCTC-3′	5′-CCTGTAACCTCTGACCTTGG-3′
IL6	5′-CTACCGCTTTCCCCACTTCAG-3′	5′-TCCTCAGCTCCTTGATGGTCTC-3′
TNFa	5′-AGCCCACGTAGTAGCAAACCC-3′	5′-TTGATGGCAGAGAGGAGGTTGA-3′
MMP2	5′-GAAGGTCAAGTGGTCCGTGT-3′	5′-CCGTACTTGCCATCCTTCTC-3′
MMP9	5-TGCCA GAGTACCTGTTCCGCTATG-3	5-TGCCACTTGAGGTCACCCTCGAA-3
TIMP1	5′-TTCTCATCGCTGGACAACTG-3′	5′-AGCGTAGGTCTTGGTGAAGC-3′
p53	5′-ATGCCTACCTCACGGGGTCT-3′	5′-AGGGTAGGGAACCAGCACCAT-3′
BNP	5′-TGC TCT TCT TGC ACC TGT-3′	5′-GCA GCT GCT GTA TCT CAG AAA-3′
CASP3	5′-GGTAGCGACAGAGTTCGAGT-3′	5′-TGAGAGGGAAGCAGAGTAACAG-3′
b-ACTIN	5′-CCATGTACGTGGCCATCCAG-3′	5′-TCTTCATGAGGTAGTCGGTCAGGTC-3′

TLR: toll-like receptor 2, 3, 4 or 8; IL6: interleukin 6; TNFa: tumor necrosis factor a; MMP: metalloproteinase 2 or 9; TIMP1: tissue inhibitor of metalloproteinase-1; p53: tumor protein 53; BNP: brain natriuretic polypeptide; CASP3: cysteinyl aspartate specific proteinase-3; b-ACTIN: β-actin.

**Table 2:** Statistical analysis of mRNA expression for each biomarker in all study groups.

Groups	C90	G30	G60	G90	C120	G120	GF120	GR120
TLR2	0.211 ± 0.078	1.120 ± 0.135 *^, a^	2.743 ± 0.422 *^, a^	3.038 ± 0.544 *^, a,^ b	0.478 ± 0.303	6.605 ± 0.800 *^, #, a, b, c^	2.768 ± 0.719 *^, #, a, d^	1.328 ± 0.195 *^, b, c, d, e^
TLR3	0.15 ± 0.04	0.2 ± 0.067	1.212 ± 0.05 *^, a^	1.273 ± 0.147 *^, a^	0.246 ± 0.097	1.252 ± 0.078 *^,^ #^, a^	0.583 ± 0.161 ^b, c, d^	0.285 ± 0.116 *^, a, b, c, d, e^
TLR4	0.19 ± 0.07	1.213 ± 0.092	5.017 ± 0.845 *^, a^	7.705 ± 0.613 *^, a, b^	0.165 ± 0.036	14.67 ± 2.925 *^, #, a, b, c^	5.277 ± 0.481 *^, #, c, d^	2.18 ± 0.886 ^b, c, d, e^
TLR8	0.188 ± 0.041	2.643 ± 0.469 *	6.98 ± 0.769 *^, a^	14.68 ± 0.876 ^*, a, b^	0.185 ± 0.04	24.47 ± 2.169 *^, #, a, b, c^	13.38 ± 1.589 *^, #, b, d^	4.962 ± 0.674 *^, #, a, b, c, d, e^
IL6	0.16 ± 0.034	2.112 ± 0.759 *	4.687 ± 0.866 *^, a^	5.768 ± 0.522 *^, a^	0.198 ± 0.047	6.324 ± 1.027 *^, #, a, b^	3.957 ± 0.389 *^, #, c, d^	3.25 ± 0.579 *^, #, b, c, d^
TNFa	0.141 ± 0.034	13.15 ± 3.073 *	14.64 ± 1.476 *	15.32 ± 2.553 *	0.13 ± 0.028	17.62 ± 2.113 *^, #, a^	13.89 ± 1.423 *^, #, d^	11.67 ± 0.834 *^, #, c, d^
MMP2	0.153 ± 0.023	32.88 ± 1.516 *	21.8 ± 2.492 *^, a^	12.57 ± 2.514 *^, a, b^	0.243 ± 0.038	7.195 ± 1.428 *^, #, a, b, c^	13.43 ± 2.279 *^, #, a^	14.06 ± 2.187 *^, #, a, b, d^
MMP9	0.206 ± 0.063	23.16 ± 1.72 *	15.37 ± 2.949 *^, a^	13.53 ± 1.408 *^, a^	0.205 ± 0.037	15.18 ± 2.002 *^, #, a^	18.03 ± 2.124 *^, #, a, c^	23.59 ± 2.1 *^, b, c, d, e^
TIMP1	0.235 ± 0.073	29.53 ± 3.81 *	36.84 ± 3.336 *^, a^	43.06 ± 3.411 *^, a, b^	0.163 ± 0.059	35.77 ± 2.033 *^, #, a, c^	22.83 ± 2.341 *, ^#, a, b, c, d^	14.83 ± 3.143 *^, #, a, b, c, d, e^
p53	0.333 ± 0.121	4.163 ± 1.498 *	7.413 ± 0.962 *^, a^	7.803 ± 1.304 *^, a^	0.348 ± 0.09	15.53 ± 1.897 *^, #, a, b, c^	6.785 ± 1.266 *, ^#, a, d^	4.42 ± 0.841 *^, b, c, d, e^
CASP3	0.13 ± 0.014	1.937 ± 0.682 *	2.552 ± 0.285 *	3.033 ± 0.665 *^, a^	0.128 ± 0.024	3.195 ± 0.258 *^, #, a^	2.65 ± 0.237 *^, #, a^	2.103 ± 0.316 *^, c, d^
BNP	0.145 ± 0.018	20.12 ± 2.34 *	23.52 ± 1.424 *^, a^	26.21 ± 1.328 *^, a, b^	0.19 ± 0.041	25.44 ± 0.998 *^, #, a^	20.63 ± 2.049 *, ^#, b, c, d^	20.4 ± 1.259 *^, b, c, d^
TIMP1/MMP2	1.53 ± 0.53	0.89 ± 0.09	1.7 ± 0.22	3.53 ± 0.67 *^, a, b, d, e^	0.67 ± 0.24	5.14 ± 1.01 *^, #, a, b, c, d, e^	1.72 ± 0.19 *^, #, c, d^	1.06 ± 0.19 ^c, d^
TIMP1/MMP9	1.24 ± 0.6	1.28 ± 0.2 *	2.46 ± 0.49 *^, a^	3.2 ± 0.37 *^, a, b^	0.8 ± 0.25	2.4 ± 0.4 *^, #, a, b, c^	1.29 ± 0.25 *^, b, c, d^	0.63 ± 0.15 *^, b, c, d^

TLR: toll-like receptor 2, 3, 4 or 8; IL6: interleukin 6; TNFa: tumor necrosis factor a; MMP: metalloproteinase 2 or 9; TIMP1: tissue inhibitor of metalloproteinase-1; TIMP1/MMP2 ratio; TIMP1/MMP9 ratio; p53: tumor protein 53; CASP3: cysteinyl aspartate specific proteinase; BNP: brain natriuretic polypeptide 3. Values are expressed as mean ± standard deviation. Significant difference (*p* < 0.05) versus C90 (*), C120 (^#^), G30 (^a^), G60 (^b^), G90 (^c^), G120 (^d^), GF120 (^e^).

## Supplementary Materials

The following are available online at https://www.mdpi.com/article/10.3390/cimb43020059/s1. Figure S1: Schematic figure representing the experimental protocol.

## References

[1] Fayad Z.A., Swirski F.K., Calcagno C., Robbins C.S., Mulder W., Kovacic J.C. (2018). Monocyte and macrophage dynamics in the cardiovascular system: JACC Macrophage in CVD Series (Part 3). J. Am. Coll. Cardiol..

[2] Goulopoulou S., McCarthy C.G., Webb R.C. (2016). Toll-like Receptors in the Vascular System: Sensing the dangers within. Pharmacol. Rev..

[3] Kaczorowski D.J., Nakao A., McCurry K.R., Billiar T.R. (2009). Toll-like receptors and myocardial ischemia/reperfusion, inflammation, and injury. Curr. Cardiol. Rev..

[4] Kawasaki T., Kawai T. (2014). Toll-like receptor signaling pathways. Front. Immunol..

[5] Kapelouzou A., Giaglis S., Peroulis M., Katsimpoulas M., Moustardas P., Aravanis C.V., Kostakis A., Karayannakos P.E., Cokkinos D.V. (2017). Overexpression of Toll-Like Receptors 2, 3, 4, and 8 Is Correlated to the Vascular Atherosclerotic Process in the Hyperlipidemic Rabbit Model: The Effect of Statin Treatment. J. Vasc. Res..

[6] Tziakas D., Chalikias G., Kapelouzou A., Tentes I., Schäfer K., Karayannakos P., Kostakis A., Boudoulas H., Konstantinides S. (2013). Erythrocyte membrane cholesterol and lipid core growth in a rabbit model of atherosclerosis: Modulatory effects of rosuvastatin. Int. J. Cardiol..

[7] Chomczynski P. (1987). Sacchi N: Single-step method of RNA isolation by acid guanidinium thiocyanate-phenol-chloroform extraction. Anal. Biochem..

[8] Livak K.J., Schmittgen T.D. (2001). Analysis of relative gene expression data using real-time quantitative PCR and the 2(-Delta Delta C(T)) Method. Methods.

[9] Hardarson H.S., Baker J.S., Yang Z., Purevjav E., Huang C.H., Alexopoulou L., Li N., Flavell R.A., Bowles N.E., Vallejo J.G. (2007). Toll-like receptor 3 is an essential component of the innate stress response in virus-induced cardiac injury. Am. J. Physiol. Heart Circ. Physiol..

[10] Cole J.E., Navin T.J., Cross A.J., Goddard M.E., Alexopoulou L., Mitra A.T., Davies A.H., Flavell R.A., Feldmann M., Monaco C. (2011). Unexpected protective role for Toll-like receptor 3 in the arterial wall. Proc. Natl. Acad. Sci. USA.

[11] Fattahi F., Russell M.W., Malan E.A., Parlett M., Abe E., Zetoune F.S., Ward P.A. (2018). Harmful Roles of TLR3 and TLR9 in Cardiac Dysfunction Developing during Polymicrobial Sepsis. BioMed Res. Int..

[12] Gao T., Zhang S.P., Wang J.F., Liu L., Wang Y., Cao Z.Y., Hu Q.K., Yuan W.J., Lin L. (2018). TLR3 contributes to persistent autophagy and heart failure in mice after myocardial infarction. J. Cell Mol. Med..

[13] Jurk M., Heil F., Vollmer J., Schetter C., Krieg A.M., Wagner H., Lipford G., Bauer S. (2002). Human TLR7 or TLR8 independently confer responsiveness to the antiviral compound R-848. Nat. Immunol..

[14] Salagianni M., Galani I.E., Lundberg A.M., Davos C.H., Varela A., Gavriil A., Lyytikäinen L.P., Lehtimäki T., Sigala F., Folkersen L. (2012). Andreakos EToll-like receptor 7 protects from atherosclerosis by constraining “inflammatory” macrophage activation. Circulation.

[15] Triantafilou K., Orthopoulos G., Vakakis E., Ahmed M.A., Golenbock D.T., Lepper P.M., Triantafilou M. (2005). Human cardiac inflammatory responses triggered by Coxsackie B viruses are mainly Toll-like receptor (TLR) 8-dependent. Cell Microbiol..

[16] Arumugam T.V., Okun E., Tang S.C., Thundyil J., Taylor S.M., Woodruff T.M. (2009). Toll-like receptors in ischemia-reperfusion injury. Shock.

[17] Spirig R., Tsui J., Shaw S. (2012). The Emerging Role of TLR and Innate Immunity in Cardiovascular Disease. Cardiol. Res. Pract..

[18] Ruysschaert J.-M., Lanez C. (2015). Role of chol and TLR. Microdomains in TLR-mediated signaling. Biochim. Biophys. Acta.

[19] Methe H., Kim J.-O., Koffler S., Nabauer M., Weis M. (2005). Statins decrease Toll-like receptor 4 expression and downstream signaling in human CD14+ monocytes. Thromb. Vasc. Biol..

[20] Hua F., Ha T., Ma J., Li Y., Kelley J., Gao X., Browder I.W., Kao R.L., Williams D.L., Li C. (2007). Protection against myocardial ischemia/reperfusion injury in TLR4-deficient mice is mediated through a phosphoinositide 3-kinase-dependent mechanism. J. Immunol..

[21] Shimamoto A., Chong A.J., Yada M., Shomura S., Takayama H., Fleisig A.J., Agnew M.L., Hampton C.R., Rothnie C.L., Spring D.J. (2006). Inhibition of Toll-like receptor 4 with eritoran attenuates myocardial ischemia-reperfusion injury. Circulation.

[22] Shishido T., Nozaki N., Yamaguchi S., Shibata Y., Nitobe J., Miyamoto T., Takahashi H., Arimoto T., Maeda K., Yamakawa M. (2003). Toll-like receptor-2 modulates ventricular remodeling after myocardial infarction. Circulation.

[23] Timmers L., Sluijter J.P., van Keulen J.K., Hoefer I.E., Nederhoff M.G., Goumans M.J., Doevendans P.A., van Echteld C.J., Joles J.A., Quax P.H. (2008). Toll-like receptor 4 mediates maladaptive left ventricular remodeling and impairs cardiac function after myocardial infarction. Circ. Res..

[24] Frantz S., Kobzik L., Kim Y.D., Fukazawa R., Medzhitov R., Lee R.T., Kelly R.A. (1999). Toll4 (TLR4) expression in cardiac myocytes in normal and failing myocardium. J. Clin. Investig..

[25] Földes G., von Haehling S., Okonko D.O., Jankowska E.A., Poole-Wilson P.A., Anker S.D. (2008). Fluvastatin reduces increased blood monocyte Toll-like receptor 4 expression in whole blood from patients with chronic heart failure. Int. J. Cardiol..

[26] Aliprantis A.O., Yang R.B., Weiss D.S., Godowski P., Zychlinsky A. (2000). The apoptotic signaling pathway activated by Toll-like receptor-2. EMBO J..

[27] Ruckdeschel K., Pfaffinger G., Haase R., Sing A., Weighardt H., Häcker G., Holzmann B., Heesemann J. (2004). Signaling of apoptosis through TLRs critically involves toll/IL-1 receptor domain-containing adapter inducing IFN-beta, but not MyD88, in bacteria-infected murine macrophages. J. Immunol..

[28] Ternacle J., Wan F., Sawaki D., Surenaud M., Pini M., Mercedes R., Ernande L., Audureau E., Dubois-Rande J.L., Adnot S. (2017). Short-term high-fat diet compromises myocardial function: A radial strain rate imaging study. Eur. Heart J. Cardiovasc. Imaging.

[29] Bartekova M., Radosinska J., Jelemensky M., Dhalla N.S. (2018). Role of cytokines and inflammation in heart function during health and disease. Heart Fail. Rev..

[30] Fujimoto S., Hartung D., Ohshima S., Edwards D.S., Zhou J., Yalamanchili P., Azure M., Fujimoto A., Isobe S., Matsumoto Y. (2008). Molecular imaging of matrix metalloproteinase in atherosclerotic lesions: Resolution with dietary modification and statin therapy. J. Am. Coll. Cardiol..

[31] Galis Z.S., Sukhova G.K., Lark M.W., Libby P. (1994). Increased expression of matrix metalloproteinases and matrix degrading activity in vulnerable regions of human atherosclerotic plaques. J. Clin. Investig..

[32] Ikeda U., Shimada K. (2003). Matrix metalloproteinases and coronary artery diseases. Clin. Cardiol..

[33] Orbe J., Fernandez L., Rodríguez J.A., Rábago G., Belzunce M., Monasterio A., Roncal C., Páramo J.A. (2003). Different expression of MMPs/TIMP-1 in human atherosclerotic lesions. Relation to plaque features and vascular bed. Atherosclerosis.

[34] Galis Z.S., Khatri J.J. (2002). Matrix metalloproteinases in vascular remodeling and atherogenesis: The good, the bad, and the ugly. Circ. Res..

[35] Antonov I.B., Kozlov K.L., Pal’tseva E.M., Polyakova O.V., Lin’kova N.S. (2018). Matrix Metalloproteinases MMP-1 and MMP-9 and Their Inhibitor TIMP-1 as Markers of Dilated Cardiomyopathy in Patients of Different Age. Bull. Exp. Biol. Med..

[36] DeLeon-Pennell K.Y., Meschiari C.A., Jung M., Lindsey M.L. (2017). Matrix Metalloproteinases in Myocardial Infarction and Heart Failure. Prog. Mol. Biol. Transl. Sci..

[37] Becirovic-Agic M., Chalise U., Daseke M.J., Konfrst S., Salomon J.D., Mishra P.K., Lindsey M.L. (2021). Infarct in the Heart: What’s MMP-9 Got to Do with It?. Biomolecules.

[38] Wang W., Schulze C.J., Suarez-Pinzon W.L., Dyck J.R., Sawicki G., Schulz R. (2002). Intracellular action of matrix metalloproteinase-2 accounts for acute myocardial ischemia and reperfusion injury. Circulation.

[39] Heymans S., Schroen B., Vermeersch P., Milting H., Gao F., Kassner A., Gillijns H., Herijgers P., Flameng W., Carmeliet P. (2005). Increased cardiac expression of tissue inhibitor of metalloproteinase-1 and tissue inhibitor of metalloproteinase-2 is related to cardiac fibrosis and dysfunction in the chronic pressure-overloaded human heart. Circulation.

[40] Takawale A., Zhang P., Patel V.B., Wang X., Oudit G., Kassiri Z. (2017). Tissue Inhibitor of Matrix Metalloproteinase-1 Promotes Myocardial Fibrosis by Mediating CD63-Integrin β1 Interaction. Hypertension.

[41] Sundström J., Evans J.C., Benjamin E.J., Levy D., Larson M.G., Sawyer D.B., Siwik D.A., Colucci W.S., Sutherland P., Wilson P.W. (2004). Relations of plasma matrix metalloproteinase-9 to clinical cardiovascular risk factors and echocardiographic left ventricular measures: The Framingham Heart Study. Circulation.

[42] Hansson J., Vasan R.S., Ärnlöv J., Ingelsson E., Lind L., Larsson A., Michaëlsson K., Sundström J. (2011). Biomarkers of extracellular matrix metabolism (MMP-9 and TIMP-1) and risk of stroke, myocardial infarction, and cause-specific mortality: Cohort study. PLoS ONE.

[43] Hansson J., Lind L., Hulthe J., Sundström J. (2009). Relations of serum MMP-9 and TIMP-1 levels to left ventricular measures and cardiovascular risk factors: A population-based study. Eur. J. Cardiovasc. Prev. Rehabil..

[44] Levine A.J. (1997). p53, the cellular gatekeeper for growth and division. Cell.

[45] Dixon L.J., Flask C.A., Papouchado B.G., Feldstein A.E., Nagy L.E. (2013). Caspase-1 as a central regulator of high fat diet-induced non-alcoholic steatohepatitis. PLoS ONE.

[46] Kehat I., Molkentin J.D. (2010). Molecular pathways underlying cardiac remodeling during pathophysiologic stimulation. Circulation.

[47] Jarai R., Kaun C., Weiss T.W., Speidl W.S., Rychli K., Maurer G., Huber K., Wojta J. (2009). Human cardiac fibroblasts express B-type natriuretic peptide: Fluvastatin ameliorates its up-regulation by interleukin-1alpha, tumour necrosis factor-alpha and transforming growth factor-beta. J. Cell Mol. Med..

[48] Tsuruda T., Boerrigter G., Huntley B.K., Noser J.A., Cataliotti A., Costello-Boerrigter L.C., Chen H.H., Burnett J.C.J. (2002). Brain natriuretic Peptide is produced in cardiac fibroblasts and induces matrix metalloproteinases. Circ. Res..

[49] Carbone S., Mauro A.G., Mezzaroma E., Kraskauskas D., Marchetti C., Buzzetti R., Van Tassell B.W., Abbate A., Toldo S. (2015). A high-sugar and high-fat diet impairs cardiac systolic and diastolic function in mice. Int. J. Cardiol..

[50] Carbone S., Lee PJ H., Mauro A.G., Mezzaroma E., Buzzetti R., Van Tassell B., Abbate A., Toldo S. (2017). Interleukin-18 mediates cardiac dysfunction induced by western diet independent of obesity and hyperglycemia in the mouse. Nutr. Diabetes.

[51] Drosatos K., Schulze P.C. (2013). Cardiac lipotoxicity: Molecular pathways and therapeutic implications. Curr. Heart Fail. Rep..

[52] Battault S., Renguet E., Van Steenbergen A., Horman S., Beauloye C., Bertrand L. (2020). Myocardial glucotoxicity: Mechanisms and potential therapeutic targets. Arch. Cardiovasc. Dis..

[53] Malmgren S., Spégel S., Danielsson A.P.H., Nagorny C.L., Andersson L.E., Dekker Nitert M., Ridderstråle M., Mulder H., Ling C. (2013). Coordinate changes in histone modifications, mRNA levels, and metabolite profiles in clonal INS-1 832/13 β-cells accompany functional adaptations to lipotoxicity. J. Biol. Chem..

[54] Cencioni C., Spallotta F., Greco S., Martelli F., Zeiher A.M., Gaetano C. (2014). Epigenetic mechanisms of hyperglycemic memory. Int. J. Biochem. Cell Biol..

[55] Keating S.T., El-Osta A. (2015). Epigenetics and metabolism. Circ. Res..

[56] Lee T.-M., Chai T.-F., Tsai C.-H. (2002). Association of pravastatin and left ventricular mass in hypercholesterolemic patients: Role of 8-150 prostaglandin fzalpha formation. Clin. Trial J. Cardiovasc. Pharmacol..

[57] Deo S.V., Rababa’h A., Altarabsheh S.E., Lim J.Y., Cho Y.H., Park S.J. (2014). Statin therapy improves long-term survival in non-ischaemic cardiomyopathy: A pooled analysis of 4500 patients. Heart Lung Circ..

[58] Gastelurrutia P., Lupon J., de Antonio M., Urrutia A., Díez C., Coll R., Altimir S., Bayes-Genis A. (2012). Statins in heart failure: The paradox between large randomized clinical trials and real life. Mayo Clin. Proc..

[59] Krum H., McMurray J.J. (2002). Statins and chronic heart failure: Do we need a large-scale outcome trial?. J. Am. Coll. Cardiol..

[60] Ward N.C., Watts G.F., Eckel R.H. (2019). Statin Toxicity. Circ. Res..

[61] Yu L., Feng Z. (2018). The Role of Toll-Like Receptor Signaling in the Progression of Heart Failure. Mediat. Inflamm..

[62] Nishimura M., Naito S. (2005). Tissue-specific mRNA expression profiles of human toll-like receptors and related genes. Biol. Pharm. Bull..

[63] Zarember K.A., Godowski P.J. (2002). Tissue expression of human Toll-like receptors and differential regulation of Toll-like receptor mRNAs in leukocytes in response to microbes, their products, and cytokines. J. Immunol..

[64] Akbal E., Koçak E., Köklü S., Ergül B., Akyürek Ö., Yılmaz F.M. (2017). Serum Toll-Like Receptor-2, Toll-Like Receptor-4 Levels in Patients with HBeAg-Negative Chronic Viral Hepatitis B. Viral Immunol..

