# Supplementary material for: A High-Cholesterol Diet Increases Toll-like Receptors and Other Harmful Factors in the Rabbit Myocardium: The Beneficial Effect of Statins
Source: Curr Issues Mol Biol. 2021 Jul 26;43(2):818–30. doi: 10.3390/cimb43020059 (PMC8928938; doi:10.3390/cimb43020059)
Supplement: Supplementary file 1 [file cimb-43-00059-s001.zip › cimb-1262448-supplementary.pdf]

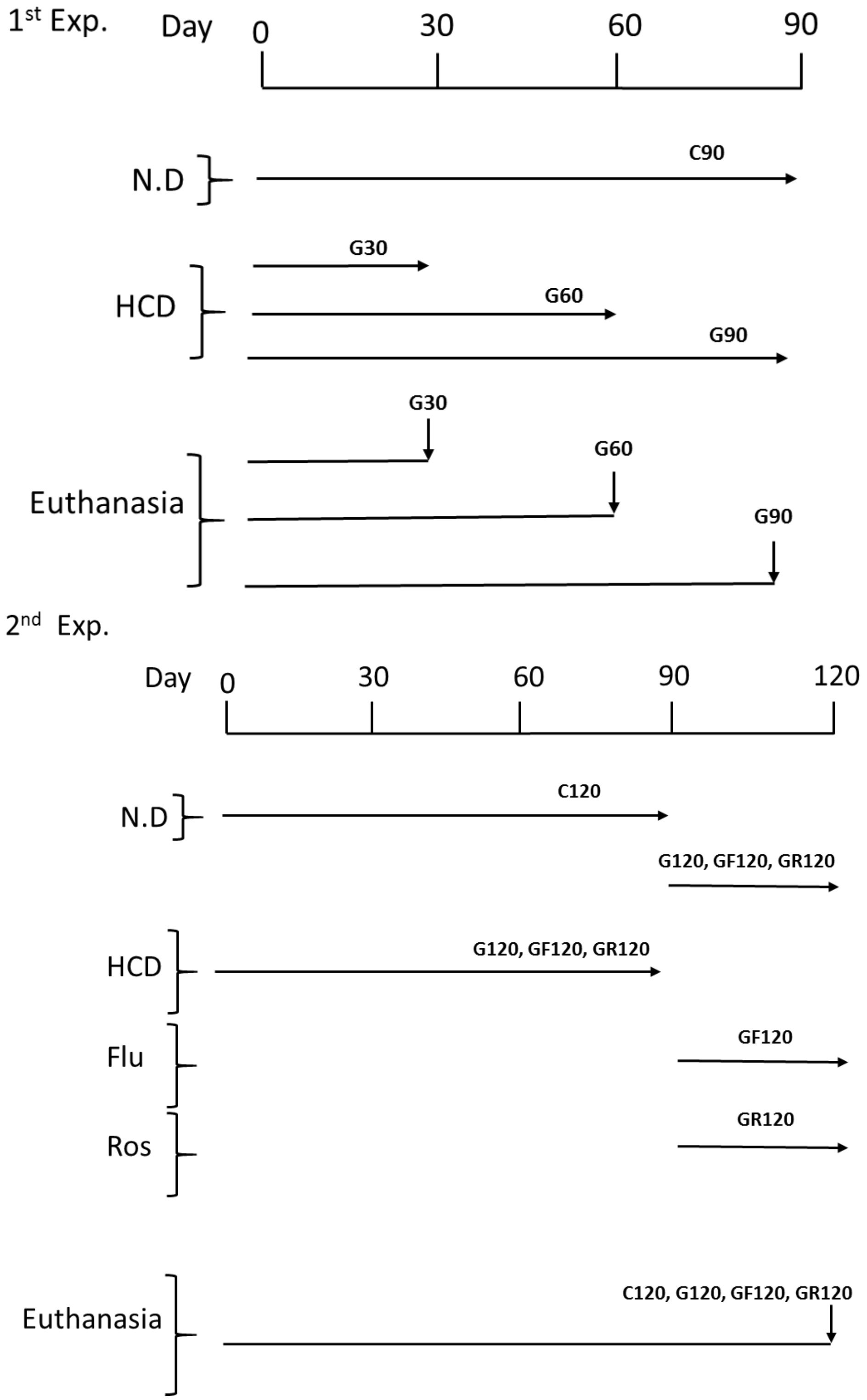

Supplementary Figure S1. Schematic figure representing the experimental protocol. First experiment describes the period of HCD intake; second experiment describes the use of the Flu and Ros. N.D, normal diet; HCD, high-cholesterol diet; Flu, fluvastatin; Ros, rosuvastatin.
